# Potential Antibiotic Resurgence: Consecutive Silver Nanoparticle Applications Gradually Increase Bacterial Susceptibility to Antibiotics

**DOI:** 10.1021/acsomega.4c09240

**Published:** 2025-01-30

**Authors:** Maria Maklakova, Luis Jesús Villarreal-Gómez, Ekaterina Nefedova, Nikolay Shkil, Roberto Luna Vázquez-Gómez, Alexey Pestryakov, Nina Bogdanchikova

**Affiliations:** † Facultad de Pedagogía e Innovación Educativa, Universidad Autónoma de Baja California, Mexicali, Baja California 21360, Mexico; ‡ Facultad de Ciencias de la Ingeniería y Tecnología, Universidad Autónoma de Baja California, Tijuana, Baja California 22260, Mexico; § Siberian Federal Scientific Centre of Agro-BioTechnologies of the Russian Academy of Sciences, Novosibirsk 630501, Russian Federation; ∥ Escuela de Ciencias de la Salud, Universidad Autónoma de Baja California, Ensenada, Baja California 22890, Mexico; ⊥ Research School of Chemistry and Applied Biomedical Sciences, Tomsk Polytechnic University, Tomsk 634050, Russian Federation; # Centro de Nanociencias y Nanotecnología, Universidad Nacional Autónoma de México, Ensenada, Baja California 22800, Mexico

## Abstract

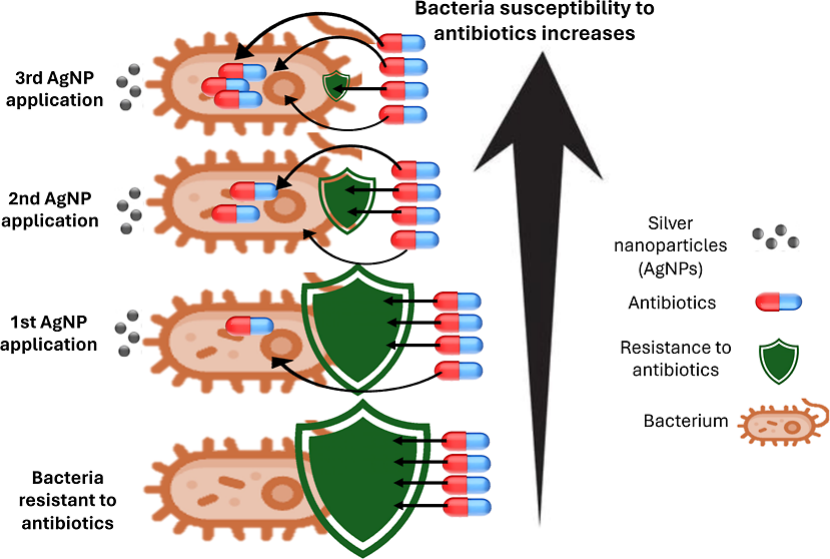

The increasing prevalence of resistant bacteria has emerged as a critical public health concern due to their ability to resist multiple antibiotics. This study aimed to investigate whether repeated treatments with silver nanoparticles (AgNPs) could gradually decrease bacterial resistance to antibiotics. The methodology involved three consecutive applications of AgNPs on six bacterial strains, followed by assessing their susceptibility to 38 different antibiotics. To our knowledge, the following three phenomena were observed for the first time. (1) During three consecutive AgNP applications, it was revealed that all the studied bacteria gradually became more susceptible to 38 antibiotics; by the end of the treatments, susceptibility had doubled for five bacteria and tripled for *Klebsiella pneumoniae* compared to the susceptibility before the first AgNP application. (2) Three consecutive AgNP treatments led to 27–47% restoration of bacterial susceptibility to antibiotics, which had already completely lost their activity before the initial AgNP application. (3) Unlike previous studies, we discovered a novel effect: the repeated AgNP applications increased the susceptibility of *Salmonella enteritidis* and *Staphylococcus aureus* to AgNPs themselves. Obtained results suggest that AgNP treatments may offer a new promising strategy to combat antibiotic resistance.

## 1 Introduction

The growing prevalence of multidrug-resistant (MDR) bacterial pathogens presents a significant threat to global public health.^1^ Among these pathogens, the ESKAPE group, including *Enterococcus faecium*, *Staphylococcus aureus*, *Klebsiella pneumoniae*, *Acinetobacter baumannii*, *Pseudomonas aeruginosa*, and *Enterobacter* species, is particularly notorious for causing severe hospital-acquired infections and demonstrating high levels of resistance to commonly used antibiotics.^2^ This resistance complicates treatment options, leading to higher morbidity, mortality, and healthcare costs.^3^

Traditional antibiotics often fail due to mechanisms such as enzymatic degradation of drugs, alterations in drug targets, efflux effects, and biofilm formation. These mechanisms enable bacteria to withstand antibiotic treatment, which demands the exploration of alternative solutions.^4^ Due to the constantly growing resistance of bacteria to antibiotics, current antimicrobial strategies are increasingly ineffective against these resilient pathogens.

Nanoparticles (NPs), especially silver nanoparticles (AgNPs), have emerged as a promising alternative to traditional antimicrobial agents, such as antibiotics and chemical disinfectants,^5^ due to their unique properties, such as their ability to generate reactive oxygen species.^6^ AgNPs exhibit broad-spectrum antimicrobial activity and can target multiple bacterial sites simultaneously, reducing the likelihood of resistance development. However, there is a potential risk that bacteria could develop resistance to nanoparticles through mechanisms like trapping them using extracellular substances and genetic mutations challenging their long-term efficacy.^7^

Recently, our group suggested one new approach consisting of using bacteria treatment with Argovit AgNPs, which decreased the resistance of bacteria to antibiotics. It was shown from in vitro and in vivo studies that AgNP treatment of cows with mastitis leads to a rise of bacterial susceptibility to 31 antibiotics on average by 19.9, 17.2, 19.4, and 12.3% for four bacteria, the most abundant in this illness: *S. aureus*, *Streptococcus dysgalactiae*, *E. coli*, and *S. epidermidis*, respectively.^8^ Five mechanisms causing the phenomenon of bacterial susceptibility growth were revealed. They include the decrease in (1) antibiotic efflux,^9^ (2) bacteria adhesion to cells,^10^ (3) bacteria antilysozyme activity,^10^ and (4) bacteria capacity of biofilm formation,^8^ caused by animal treatment with AgNPs. A logical continuation of these studies was to clarify if the bacterial susceptibility growth after bacteria treatment with AgNPs occurs only once (after the first treatment with AgNPs) or if this effect gradually increases after every successive treatment with AgNPs. So, this work is devoted to finding the answer to this question.

## 2 Materials and Methods

### 2.1 Treatment Formulations

The list of the applied antibiotics included amikacin, neomycin, tobramycin, streptomycin, gentamicin, kanamycin, ciprofloxacin, enrofloxacin, norfloxacin, ofloxacin, tetracycline, doxycycline, oxytetracycline, carbenicillin, ampicillin, benzylpenicillin, amoxicillin, ticarcillin, cefotaxime, cefazolin, cephalexin, cefuroxime, cephalexin, cefaclor, ceftriaxone, erythromycin, tylosin, azithromycin, lincomycin, rifampicin, levomycetin (chloramphenicol), trimethoprim, imipenem, colistin, polymyxin, furazolidone, and furagin (FSBI Research Institute of Epidemiology and Microbiology named after Pasteur, St. Petersburg, Russia). Ceftiofur from Bioanalyse Medical Supplies Industry and Trade Co. Ltd., Ankara, Turkey, was used. So, the resistance to 38 antibiotics, the representatives of 8 groups: 1. aminoglycosides, 2. fluoroquinolones, 3. tetracyclines, 4. penicillins, 5. cephalosporins, 6. macrolides, 7. others, 8. polymyxins, and 9. nitrofurans, was studied.

#### 2.1.1 Argovit-C AgNP Formulation

The formulation produced by the Vector-Vita Research and Production Center, Novosibirsk, Russia, as a veterinary drug, was provided by Dr. Vasily Burmistrov. Argovit-C AgNPs is a stable aqueous suspension of silver nanoparticles (which includes metallic silver and stabilizer) with a concentration of 200 mg/mL (20% by weight). The concentration of metallic silver (active component) is 12 mg/mL (1.2 wt %). The concentration of the stabilizer, containing polyvinylpyrrolidone (1/3) and collagen hydrolysate (2/3), is 188 mg/mL (18.8 wt %).

**Figure 1:**
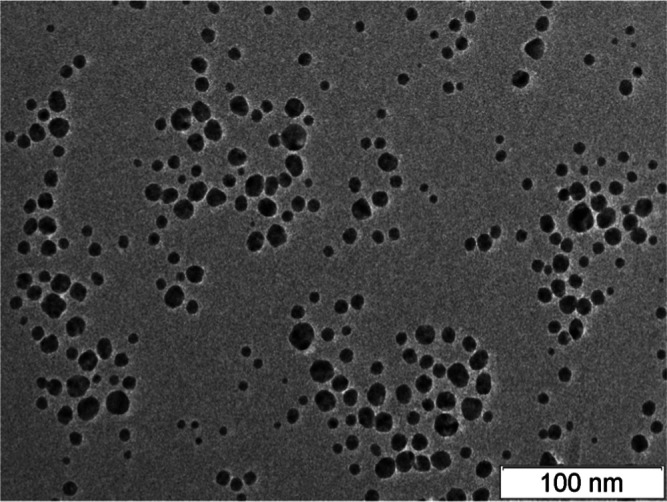
Electron microscopic image of AgNPs deposited from the original solution diluted with distilled water (1:20) and processed with ultrasound with a frequency of 35 kHz. The image was made on a JEOL JEM-2010 microscope (JEOL, Japan).

The method of the synthesis of Argovit AgNPs includes the following steps. First, a solution of the mixture of protein hydrolysate (2/3) and polyvinylpyrrolidone (1/3) with a total concentration of 18.8 wt % was prepared. Then, silver nitrate solution necessary to reach 1.2 wt % of AgNPs (12 mg/mL of metallic silver) was prepared and stirred at room temperature until completely dissolved. The resulting silver salt solution was added to a vessel with the appropriate amount of stabilizer solution, intensively mixed, and exposed to an accelerated electron beam (voltage 30 kV) of high-energy (2–2.5 MeV) electrons with an absorbed dose of 15 kGy generated on a linear accelerator ILU-10 (Institute of Nuclear Physics, Novosibirsk, Russia). Electron beam treatment led to the formation of stable AgNPs. AgNPs are represented in spheroids (Figure 1) with sizes lying in the interval 2–50 nm with an average of 11.76 ± 5.53 nm.

Identification of AgNPs was made with UV–visible spectroscopy by the absorption band in the interval 420–460, typical for AgNPs. The average hydrodynamic diameter of AgNPs (silver nuclei together with is 142.6 ± 0.241 nm). Potential zeta is +9.4 ± 6.28 mV. Detailed characterization of physicochemical properties of AgNP formulation is described in ref (11).

### 2.2 Bacterium Strains

*S. aureus* ATCC 25953, *K. pneumoniae* ATCC 13883, and *P. aeruginosa* ATCC 27853 (belonging to the ESKAPE group) and *S. pyogenes* ATCC 19615, *S. enteritidis* ATCC 13076, and *E. coli* ATCC 25922 were studied. Previously obtained results of the microbiological study of milk samples from 200 cows with serous mastitis showed that these bacteria are predominant in cows with mastitis in our experiments.

### 2.3 MIC and MBC Determination

**Figure 2:**
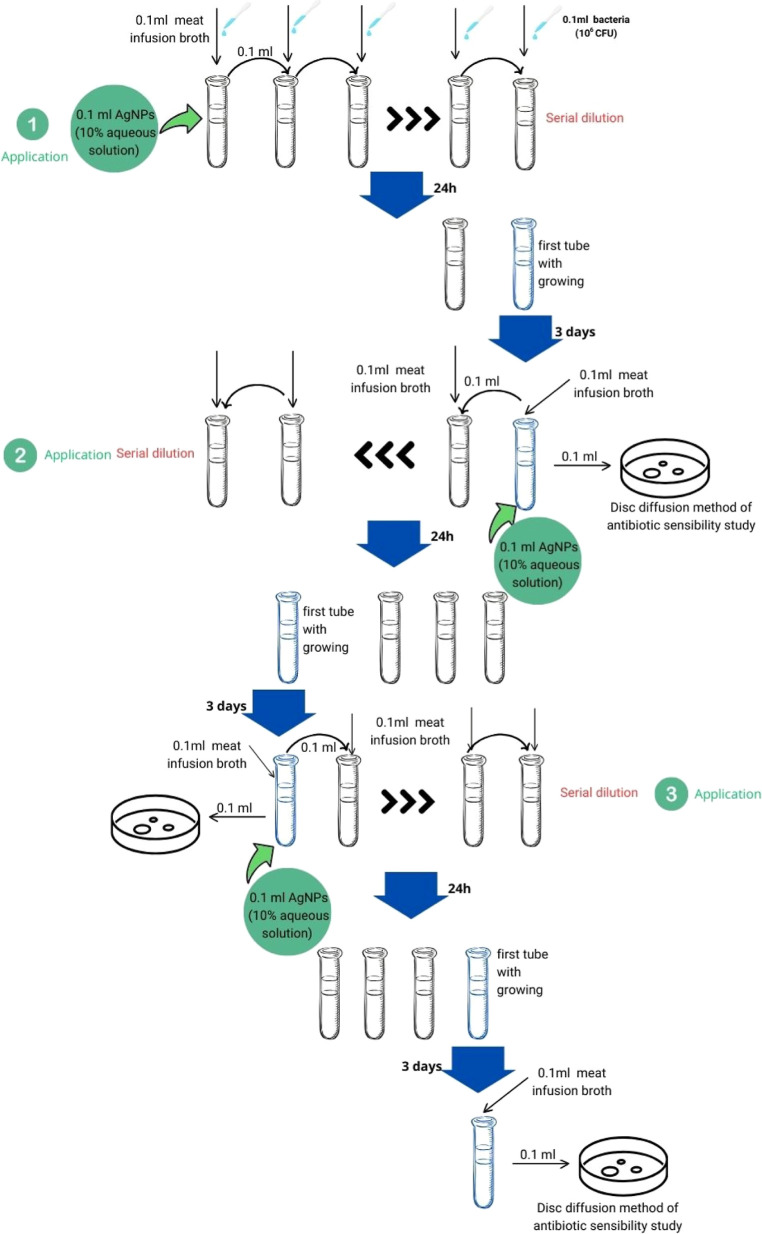
Diagram of the stages of the experiment.^13^

Minimum inhibitory concentration (MIC) and minimum bactericidal concentration (MBC) for silver nanoparticles were determined with the broth macrodilution technique,^12^ and it was repeated three times. The diagram of the stages of the experiment is presented in Figure 2. Experiments were carried out with bacteria concentration adjusted to 10^6^ CFU/ml (following the 0.5 McFarland standard) in MP broth. Inoculated broth and inoculated broth with a microbial suspension were used as negative and positive controls, respectively. Meat–peptone broth (LLC Scientific Research Center of Pharmacotherapy LLC, St. Petersburg, Russia) and Mueller–Hinton agar (Biomedia LLC, St. Petersburg, Russia) were used.

All concentrations in this work refer to the concentration of metallic silver (the active component of AgNPs). The minimum inhibitory concentration (MIC) was defined as the lowest concentration of nanoparticles that prevented visible microbial growth. The tubes were examined for turbidity before and after incubation to confirm the MIC. Following MIC determination, 50 μL samples from tubes without visible bacterial growth were plated on meat–peptone agar and incubated for 24 h at 37 °C. The minimum bactericidal concentration (MBC) was determined as the lowest concentration of silver nanoparticles that killed 99.9% of the bacterial population. The presence or absence of bacterial growth on the agar plates, both before and after incubation, was used to identify the MBC.

### 2.4 Biofilm Formation

Biofilm formation was analyzed by a crystal violet assay.^14^ The biomass of the studied cultures was seeded with a standard bacteriological loop in 4 mL of nutrient broth and cultured at 37 °C for 18–24 h. Next, 1.8 mL of meat–peptone broth and 0.2 mL of bacterial suspension (1.5 × 10^6^ CFU/ml) were added to every well of the 96-well tablet (0.5 McFarland standard). After incubation for 24 h, planktonic microorganisms were removed from each well, and the content of the wells was washed with distilled water. After that, 125 μL of 0.1% crystal violet solution was added for 15 min at room temperature for staining. Then, the solution was removed, and the well content was washed with distilled water. The tablet was dried in air, and 200 μL of 95% ethyl alcohol was introduced into all wells. The tablet was incubated for 15 min at room temperature, and then 125 μL of the remaining alcohol extract was transferred to a clean 96-well plate for the next step of the experiment.

The optical density of the final suspensions was measured on a STAT FAX 2100 spectrophotometer (Awareness Technology, Inc., Palm City, FL, USA) at 490 nm. The measured optical density corresponded to the degree of biofilm formation. The biofilm formation activity (BFA) index was calculated with the formula
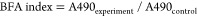
where A490_experiment_ is the optical density of the experimental sample at wavelength 490 nm and A490_control_ is the optical density of the negative control. Commercial strain with broth was used as a positive control; broth was applied as a negative control. More details of the method are presented in our previous article.^8^

### 2.5 Statistical Analysis

The results were statistically processed by using the methods of parametric and nonparametric analysis. The accumulation, correction, systematization of the initial information, and visualization of the results were carried out with GraphPad Software 9.0, San Diego, CA, USA. Statistical analysis was carried out using the STATISTICA 13.3 program (StatSoft, Inc., Tulsa, OK, USA).

## 3 Results

### 3.1 Minimal Bactericidal and Inhibitory Concentrations and Resistance to AgNPs

**Figure 3:**
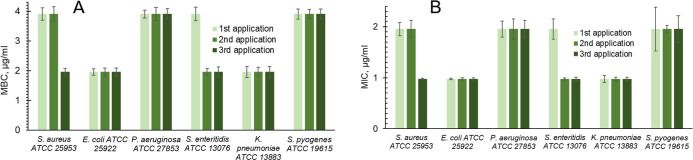
Minimal bactericidal concentration (A) and minimal inhibitory concentration (B) after each of three applications of AgNPs.

Figure 3 presents AgNP MIC and MBC concentrations after each of three applications of AgNPs. The graph of the MIC after three incubations with AgNPs (Figure 3B) shows the same dynamic as for MBC (Figure 3A). For *S. aureus* and *S. enteritidis*, a decrease from 1.95 to 0.98 μg/mL was observed after the second and the first applications, respectively. For all other four bacteria, either MIC or MBC were constant after each of three applications of AgNPs.

**Figure 4:**
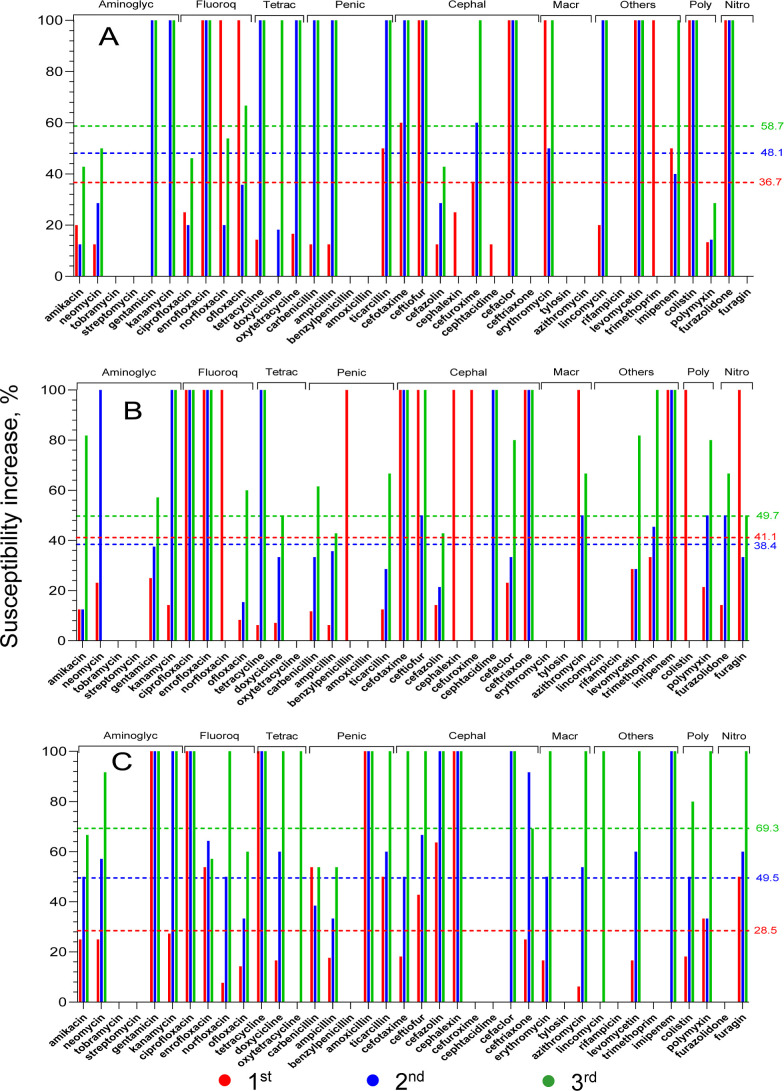

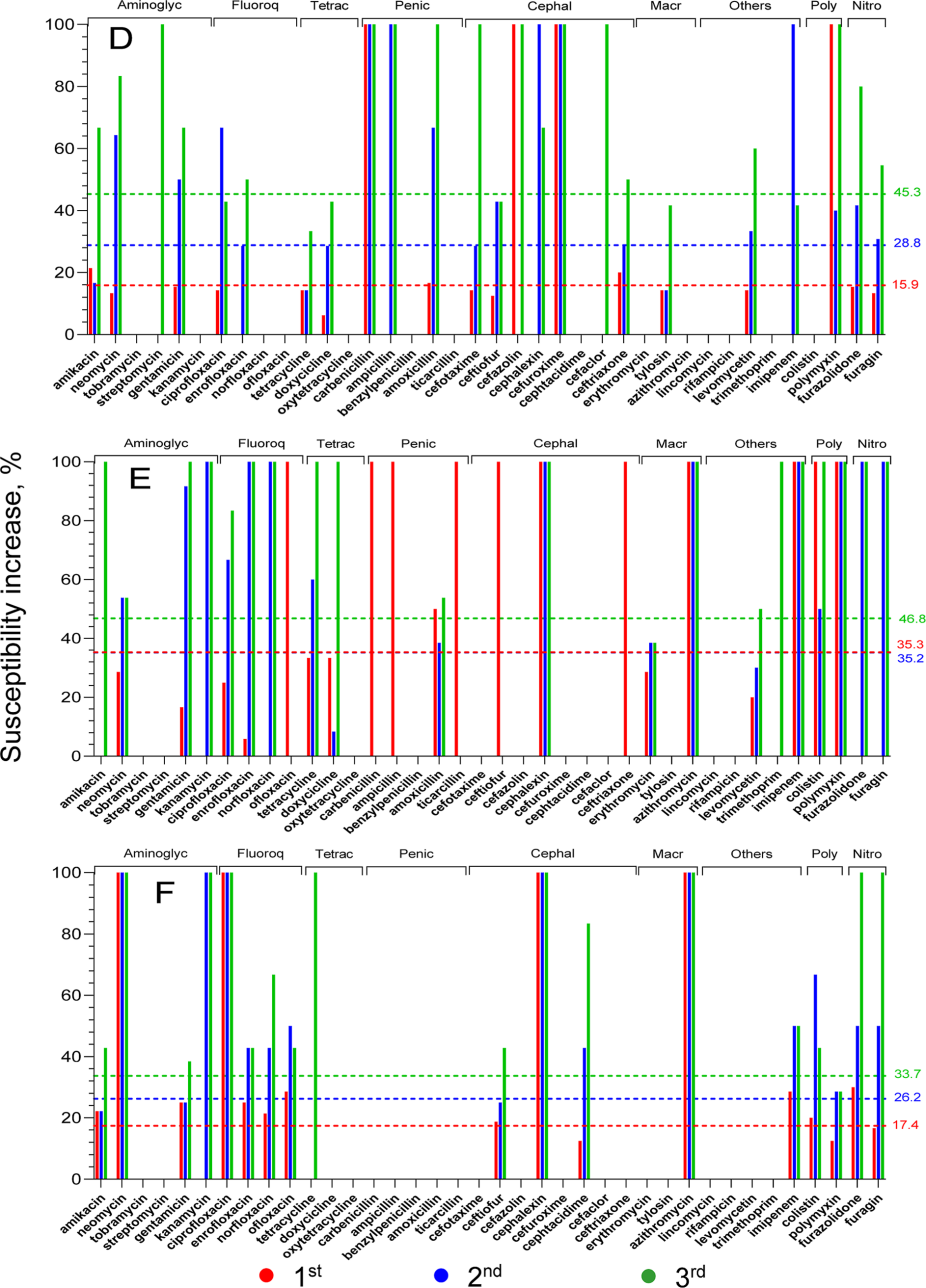
Changes of antibiotic susceptibility after each of three AgNP applications: (A) *S. pyogenes*, (B) *S. enteritidis*, (C) *S. aureus*, (D) *E. coli*, (E) *K. pneumoniae*, and (F) *P. aeruginosa*. The susceptibility growth after the first, second, and third AgNP applications are presented as red, blue, and green columns, respectively. The average (for 38 antibiotics) susceptibility growth for every AgNP application is presented with a dotted line of the corresponding color.

Figure 4A–F presents changes in the susceptibility to 38 antibiotics after three sequential AgNP applications for all six studied bacteria. Studied antibiotics belong to eight groups marked at the top of each figure (Figure 4A–F). The percentages of the increase in susceptibility after the first, second, and third AgNP applications are presented as red, blue, and green columns, respectively (Figure 4). AgNP applications (Figure 4) were carried out with AgNPs at concentrations corresponding to their MBCs measured in previous experiments (Figure 3A).

**Figure 5:**
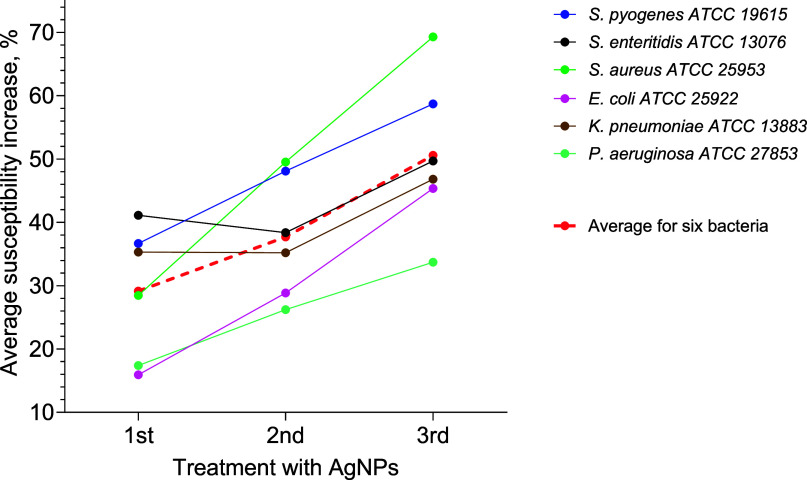
Average (for 38 antibiotics) growth of susceptibility for each of three sequential AgNP applications: *S. pyogenes* – blue, *S. Enteritidis* – black, *S. aureus* – green, *E. coli* – pink, *K. pneumonia* – brown, and *P. aeruginosa* – light green.

Figure 5A–F illustrates that the average susceptibility of each of the studied bacteria to 38 antibiotics increased for every AgNP application, as presented with dotted lines. Figure 5 illustrates these average susceptibility changes for all six studied bacteria after each of three consecutive AgNP applications.

To simplify the detailed analysis of Figures 4 and 5, the percentages (%) of antibiotics with different behaviors after three consecutive AgNP applications for six studied bacteria are presented in Table 1. Table 1 presents 228 cases of 12 behavior types calculated for 6 bacteria with the use of 38 antibiotics (6 bacteria × 38 antibiotics = 228 cases). This table shows that the most typical behaviors for studied antibiotics were (1) growth of the susceptibility to antibiotics, (2) remaining at 0%, and (3) remaining at 100% growth with the increasing number of AgNP applications. Further analysis of the table will be done in the Discussion section.

**Table 1:** Number of Antibiotics with Different Behaviors after Three Consecutive AgNP Applications for Six Studied Bacteria

	percentage of antibiotics (%)											
	which with AgNP application numbers						registration of susceptibility only after specific applications					
bacterium	1) remains 0%	2) remains 100%	3) grows	4) drops	5) goes through max	6) goes through min	7) first	8) second	9) third	10) first + second	11) second + third	12) first + third
S. pyogenes ATCC 19615	8	**6**	**12**	0	0	6	**3**	0	0	0	**3**	0
S. enteritidis ATCC 13076	8	**5**	**15**	0	0	3	**5**	0	0	1	**1**	0
S. aureus ATCC 25953	9	**5**	**17**	0	2	1	0	0	2	0	**2**	0
E. coli ATCC 25922	**14**	2	12	0	1	2	0	0	2	0	**4**	1
K. pneumoniae ATCC 13883	**12**	4	7	0	0	3	**6**	0	2	0	**4**	0
P. aeruginosa ATCC 27853	**20**	4	10	0	2	0	0	0	1	0	**1**	0
total number of antibiotics with the specific behavior	71	26	73	0	5	15	14	0	7	1	15	1

The total gain of the consequent AgNP application (from the susceptibility of the first control to the susceptibility of bacteria after the third AgNP application) is demonstrated in Table 2. The most impactful change of 195.8% increase was observed for *K. pneumoniae*, and the lowest was for *S. aureus* (89.42%) and *P. aeruginosa* (96.05%). So, all studied bacteria, thanks to three AgNP applications, decreased their antibiotic resistance approximately twice and *K. pneumoniae* three times.

**Table 2:** Difference between Average Susceptibility after the Third Application and Average Susceptibility before the First Application for 38 Antibiotics (Including Antibiotics with Null Activity)

bacteria	average change of antibiotic susceptibility from the first control to the third application, %
S. pyogenes ATCC 19615	125.70
S. enteritidis ATCC 13076	100.87
S. aureus ATCC 25953	89.42
E. coli ATCC 25922	115.58
K. pneumoniae ATCC 13883	195.80
P. aeruginosa ATCC 27853	96.05

The difference between average susceptibility after the third application and average susceptibility after the first application for 38 antibiotics (including antibiotics with null activity) is presented.

It was of interest to pay special attention to the consideration of the number of antibiotics which initially had zero susceptibility but after the third application of AgNPs developed sensitivity (Table 3):
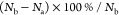
where *N*_b_ is the number of antibiotics with zero susceptibility before the first AgNP application and *N*_a_ is the number after the third AgNP application. Their quantity for six bacteria varies between 26.92 (for *P. aeruginosa*) and 47.06% (for *S. aureus*). These results indicate that on average, for 39% of the antibiotics with zero initial susceptibility, after the third application, the susceptibility emerged (Table 3).

**Table 3:** Quantity of Antibiotics with Zero Activity before the First Treatment with AgNPs and after the Third Treatment and Their Difference

	a quantity of antibiotics with zero activity			
bacteria	before the first AgNP application	after the third AgNP application	difference	% difference
S. pyogenes ATCC 19615	22	12	10	–45.45
S. enteritidis ATCC 13076	22	14	8	–36.36
S. aureus ATCC 25953	17	9	8	–47.06
E. coli ATCC 25922	24	14	10	–41.67
K. pneumoniae ATCC 13883	29	18	11	–37.93
P. aeruginosa ATCC 27853	26	19	7	–26.92
**For all bacteria**	**140**	**86**	**54**	**Average −39**

**Figure 6:**
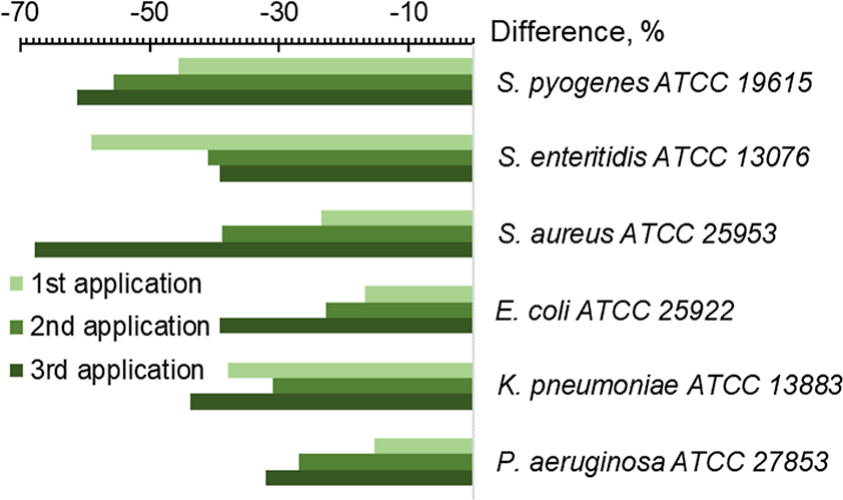
Difference of average quantity of antibiotics with “0” activity after each of three applications of AgNPs, %.

Figure 6 illustrates the percentage of the change in the number of antibiotics with zero activity after the first, second, and third AgNP applications for every studied bacterium.

For four bacteria (*S. pyogenes*, *S. aureus*, *E. coli*, and *P. aeruginosa*), susceptibility recovery increases with three consecutive AgNP applications (Figure 6). However, for *S. enteritidis* and *K. pneumoniae*, recuperation after the first application is high, but after the next applications, it drops or goes through a minimum, respectively. So, the behavior of these two bacteria was very different from the behavior of the other four bacteria (Figure 6).

### 3.2 Biofilm Formation

**Figure 7:**
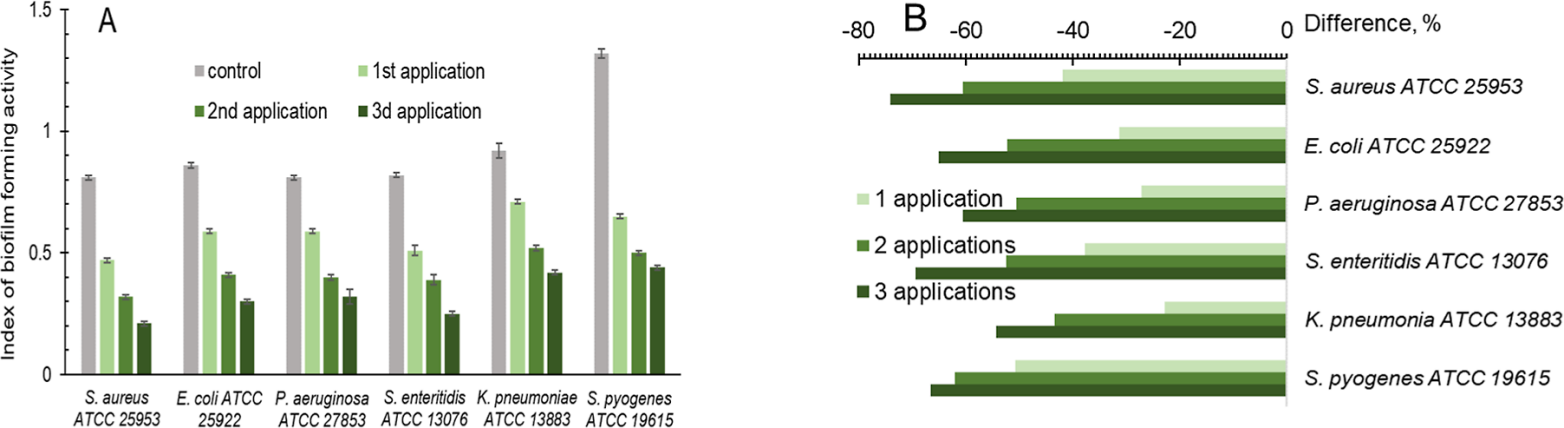
(A) Index of biofilm formation activity before and after application of AgNPs. (B) Difference of index of biofilm-forming activity before and after AgNP applications. The gray column is data before the first AgNP application; green colors with increasing intensity correspond to the first, second, and third AgNP applications.

Figure 7A presents the index of biofilm formation activity before the first AgNP addition and after the first, second, and third AgNP applications for six bacteria. The character of the change in BFA is similar for all six bacteria.

Figure 7B illustrates the difference in the change in BFA after every three AgNP applications. After the first application, the BFA index was reduced by 22.8–50.8%; after the second, it decreased by 43.5–62.1%, and after the third use, it dropped by 54.3–74.1%.

## 4 Discussion

Three of the six bacteria studied in this work (*S. aureus*, *P. aeruginosa*, and *K. pneumoniae*) are in the list of ESKAPE^15^ of bacteria with multiple antibiotic resistance and high virulence in humans. As can be seen in Figure 5, the average increase of susceptibility to 38 antibiotics for each of the six bacteria first can be divided into two groups. Two bacteria with maximum susceptibility growth (*S. aureus* and *S. pyogenes*) present the first group, and the other four bacteria (*S. enteritidis*, *E. coli*, *K. pneumoniae*, and *P. aeruginosa*) with moderate susceptibility growth present the second group. The first group, which includes *S. aureus* and *S. pyogenes*, consists of Gram-positive bacteria, while the other four are Gram-negative. Gram-positive bacteria have single-layered cell walls, which may allow compounds to penetrate more easily compared to the multilayered walls of Gram-negative bacteria, which have additional layers beyond the peptidoglycan layer. However, despite the wider cell wall of Staphylococcus spp. and Streptococcus spp. compared to the Gram-negative bacteria used, it is likely that the higher susceptibility observed in *S. aureus* and *S. pyogenes* is due to the differences in cell wall structure.

Table 1 presents the percentage of antibiotics with different behavior types after three consecutive AgNP applications for the six studied bacteria. These behavior types include a group with constant zero susceptibility (1), susceptibility increasing by 100% (2), and susceptibility increasing by less than 100% (3). These first three behavior types present 75% of all cases (Table 1). For *S. aureus*, *S. pyogenes*, and *S. enteritidis*, the ratios of antibiotic quantity with growing susceptibility (sum of antibiotics from the second and the third groups) to an antibiotic quantity with zero growing susceptibility (the first group, Table 1) are 2.3–2.5, which is 2–3 times higher than for the other three bacteria, for which this ratio lies between 0.7 and 1.0 (Table 4). These ratios can explain the higher positions for *S. aureus*, *S. pyogenes*, and *S. enteritidis* in Figure 5. In Figure 5, their data predominantly are located higher than the straight line representing the average values for six bacteria (red dotted line). This ratio for *P. aeruginosa* is minimal (0.7, Table 4), which correlates with the lowest position for this bacterium in Figure 4.

**Table 4:** Calculation of the Ratio of the Number of Antibiotics with Growing Susceptibility to the Number of Antibiotics with Zero Susceptibility for Studied Bacteria

bacterium	(1) remaining 0%	(2) 100% growth + (3) increasing	(2 + 3)/(1)
S. pyogenes ATCC 19615	8	18	2.3
S. enteritidis ATCC 13076	8	20	2.5
S. aureus ATCC 25953	9	22	2.4
E. coli ATCC 25922	14	14	1.0
K. pneumoniae ATCC 13883	12	11	0.9
P. aeruginosa ATCC 27853	20	14	0.7

As it was shown in our previous work for unique AgNP application, a decrease in the BFA index after AgNP application can be one of the reasons for the gradual increase of the bacterial susceptibility to antibiotics after each of the three AgNP applications.^8^ After the first AgNP application, the BFA index of six bacteria was reduced by 22.8–50.8%; after the second use, it decreased by 43.5–62.1%, and after the third use, it dropped by 54.3–74.1% (Figure 7B). So, the results of the present work showed that every new AgNP application led to a more significant drop in bacterium biofilm capacity (Figure 6).

It should be noted that the shape of the graphs in Figure 5 for two bacteria, *S. enteritidis* and *K. pneumoniae,* differs from the shape of the graphs for other bacteria. For *S. enteritidis*and *K. pneumoniae*, the susceptibility was practically constant after the first and second AgNP applications, and it increased only after the third AgNP application (Figure 5). While other graphs present straight lines with constant growth. We can hypothesize that *S. enteritidis* and *K. pneumoniae* can form polysaccharide capsules^16,17^ and, in such a way, protect themselves from the intensive impact of AgNPs after the second application.

In Table 5, the results on bacteria resistance to AgNPs obtained in the present article (Figure 3) are compared with the literature data.

**Table 5:** Bacteria Resistance to AgNPs after Various AgNP Applications^a^

nanoparticles	preparation method	characteristics (morphology, aggregation)	np size (nm)	ζ potential (mV)	application frequency	applied bacteria	observation	resistance	conc. (μg/mL)	ref
AgNPs S0	commercial	aggregate formation	10–30	45 mV	repeated (3×)	E. coli, K. pneumoniae, Enterobacter cloacae, S. aureus	variants selected after repeated exposure showed altered sensitivity	increased	0.5, 1, 4	(18)
AgNPs S1	chemical reduction with TiO_2_ (TiO_2a_/Ag^0^)		10–20	–35		E. coli	developed resistance			
AgNPs S2			20–40	–30		K. pneumoniae				
AgNPs S3			30–50	–25		Enterobacter cloacae				
AgNPs S4			40–60	–20		S. aureus				
AgNPs S5			50–70	–15		E. coli				
AgNPs S6			60–80	–10		K. pneumoniae				
AgNPs S7	commercial		70–90	–5		Enterobacter cloacae				
AgNPs	reduction of the complex cation [Ag (NH_3_)_2_] ^+^ by d-maltose	spherical	28	not specified	repeated (20×)	E. coli CCM 3954	MICs increased more significantly for resistant strains compared to their nonresistant parent strains	increased	0.108–0.432	(19)
						P. aeruginosa CCM 3955				
						E. coli 013				
AgNPs	s	spherical	∼26	–8.9	repeated (8×)	E. coli K-12 MG1655	motile strain	increased	0.0004–0.2	(20)
					repeated (13×)	E. coli K-12 JW1881	nonmotile strain	increased	0.0004–0.2	
AgNPs	reduction with ampicillin	spherical	9–20	+33.42	repeated (53×)	E. coli	no developed resistance	constant	18.75	(21)
						S. aureus			9.375	
						ampicillin-resistant E. coli			10	
						ampicillin-resistant S. aureus			3	
						K. pneumoniae (MDR)			28.12	
						P. aeruginosa (MDR)			20	
CIP/AgNPs	commercial	spherical	11.78 ± 2.71	–10	repeated (7×)	P. aeruginosa	no developed resistance	constant	3.77	(22)
AgNPs	biological reduction with native maize starch	quasispherical	18.21 ± 9.28	–15.27	repeated (40×)	E. coli MG1655	developed resistance	increased	5.5 ± 1.74	(23)
PVP-coated AgNPs	commercial	spherical	23.4 ± 4.9	–17.7 ± 1.0	repeated (20×)	K. pneumoniae K1–K28	resistance with MIC first increased and then stabilized	constant	93.1 ± 193.3	(24)
citrate-coated AgNPs	commercial	spherical	10	*–0.3 to +0.35*	repeated (250×)	E. coli K-12 MG1655 (ATCC #47076)	bacteria can easily evolve resistance to AgNPs, and this occurs by relatively simple genomic changes	increased	50	(25)
PVP-coated AgNPs						E. coli K-12 MG1655 (ATCC #47076)		increased	50	
PVP- coated AgNPs	reduction by electron beam of high energy	spherical	2–50	+9.4 ± 6.28	repeated (3×)	S. pyogenes ATCC 19615	no developed resistance to AgNPs themselves	constant	1.95	present work
						K. pneumoniae ATCC 13883			0.98	
						P. aeruginosa ATCC 27853			1.95	
						E. coli ATCC 25922			0.98	
						S. enteritidis ATCC 13076	resistance to AgNPs themselves decreases	decreased	1.95	
						S. aureus ATCC 25953			1.95	

a CIP: ciprofloxacin; PVP: poly(vinylpyrrolidone).

Five of eight articles showed that different species of bacteria can develop resistance to 13 AgNP formulations^18−20,23,25^ (Table 5). In the case of 3 AgNP formulations, after repeated AgNP application, susceptibilities to AgNPs were constant.^18,24,25^ Analysis of the physicochemical properties of different AgNP formulations, their concentrations, and methods of preparation (Table 5) did not allow us to identify the AgNP properties or experimental conditions that led to different susceptibility changes (constant or decreasing) after multiple AgNP applications.

In the present work, the constant susceptibility to AgNP after repeated AgNP applications was also revealed for four bacteria (Table 5). However, in contrast to all AgNP formulations of all works published previously, in the present work, the effect of the increase of susceptibility of *S. enteritidis* and *S. aureus* to AgNPs during three AgNP applications was revealed (Table 5). As far as we are aware, this effect of the increase in bacterial susceptibility to AgNPs after repeated AgNP applications was reported for the first time. Probably, this difference can be explained by the peculiarity of the physicochemical properties of the AgNP formulation that we used. However, more experiments are needed to confirm this assumption. Also, experiments with a quantity of AgNP applications higher than three and with other bacterial strains should be carried out in the future.

It should be noted that in our work, the multiple applications of AgNPs were applied at very low concentrations (0.98–1.95 μg/mL), while in some works presented in Table 5, the AgNP concentrations reached 200, 320, and 500 μg/mL. In our case, at 0.98–1.95 μg/mL, bacteria grew very slowly, and we were forced to make 3 day intervals between AgNP applications so that a mass of bacteria sufficient for the experiments could grow. In the works of other groups, on the contrary, since during multiple AgNP applications resistance to AgNPs developed, the concentration of AgNPs in each next AgNP application had to be constantly increasing (Table 5).

In our previous works, several steps to uncover the causes of the phenomenon of the increase of bacterial susceptibility to antibiotics after AgNP treatments in vitro and in vivo have been taken. We showed that this effect can be explained by the decrease in the bacteria efflux effect, their adhesion to the cells, their antilysozyme activity, and the biofilm formation activity index.^8−10,26^ The present study is one more step toward answering the question: “Can AgNP treatment of bacteria arrest the evolution of antibiotic resistance?”. All our results published by our group earlier and described in this paper show that AgNP treatment can not only arrest but also decrease bacterial resistance to antibiotics.

A search for publications devoted to the study of the effect of AgNP treatment on the susceptibility of bacteria to antibiotics led to the finding of only one article.^27^ Results of this work showed that pre-exposure of *E. coli* and *S. aureus* to one AgNP sublethal dose led to increased resistance of these bacteria toward antibiotics with IC_50_ elevated by 3–13 times for the minimal inhibitory concentration and by 2–8 times for minimal biocidal concentration. So, the results of this work^27^ made for two bacteria, three antibiotics, and one AgNP application are opposite to the results obtained in the present work for six bacteria, 38 antibiotics, and three AgNP applications.

Reference (22) is the work with the topic close to the topic of the present article. In this work,^22^ the influence of 7-fold applications (alternating AgNP with ciprofloxacin) on the susceptibility of *P. aeruginosa* to AgNPs and ciprofloxacin was studied. These 7-fold “AgNPs-antibiotic” alternative applications led to maintaining constant *P. aeruginosa* susceptibility to AgNPs and an extremely slow decrease of *P. aeruginosa* susceptibility to ciprofloxacin. The experimental designs of ref (22) and our work are different. We applied three consecutive AgNP applications, whereas in ref (22), seven “AgNPs-ciprofloxacin” alternating applications were used. In ref (22), the study was done for one bacterium and one antibiotic, while in the present work, bacterial susceptibilities to 38 antibiotics for each of the six bacteria were studied. So, data obtained in these two published works^21,28^ allow us to hypothesize that restoring antibiotic effectiveness produced by AgNP treatment observed until now only for Argovit AgNP formulation is the property of specific Argovit AgNP formulation. More systematic studies with other AgNP formulations, which will be done in the future, will show the correctness of this hypothesis. In the future, the investigation of the change in bacterial susceptibility to antibiotics after treatments made several sequential times with (1) antibiotics and (2) other formulations of AgNPs will extend the study of the effects revealed in this work.

The results obtained in the present work provide innovative knowledge in the nanomedicine area with the potential to be used in the fight against bacterial diseases and opening a renaissance for antibiotics for which pathogenic bacteria have already developed resistance.

## 5 Conclusions

This systematic study focuses on examining changes in bacterial susceptibility to 38 antibiotics and Argovit-C AgNPs following three consecutive AgNP treatments across six bacterial species (*S. aureus*, *K. pneumoniae*, *P. aeruginosa*, *S. pyogenes*, *E. coli*, and *S. enteritidis*). To the best of our knowledge, the following three phenomena were revealed for the first time. During three consecutive Argovit-C AgNP applications, it was revealed (1) that all the studied bacteria gradually became more susceptible to 38 antibiotics; by the end of the treatments, susceptibility had doubled for five bacteria and tripled for *K. pneumoniae* compared to the susceptibility before the first AgNP application; (2) restoration of susceptibility of six bacteria to 26.9–47.1% of antibiotics, which had already completely lost their activity before the first AgNP application; and (3) the increase of susceptibility of *S. enteritidis* and *S. aureus* to AgNPs themselves (in contrast to the results of previous publications).

## Data Availability

Data are contained within the article.

## Abbreviations

AgNP: silver nanoparticle
ATCC: American Type Culture Collection
BFA: biofilm formation activity
CFU: colony-forming unit
CLSI: Clinical and Laboratory Standards Institute
CIP: ciprofloxacin
ESKAPE: *Enterococcus faecium*, *Staphylococcus aureus*, *Klebsiella pneumoniae*, *Acinetobacter baumannii*, *Pseudomonas aeruginosa* Enterobacter spp
PVP: poly(vinylpyrrolidone)
MBC: minimum bactericidal concentration
MDR: multidrug-resistant
MIC: minimum inhibitory concentration

## References

[ref1] Ravi K.; Singh B. ESKAPE: Navigating the Global Battlefield for Antimicrobial Resistance and Defense in Hospitals. Bacteria 2024, 3 (2), 76–98. 10.3390/bacteria3020006.

[ref2] Pandey R.; Mishra S. K.; Shrestha A. Characterisation of ESKAPE Pathogens with Special Reference to Multidrug Resistance and Biofilm Production in a Nepalese Hospital. Infect. Drug Resist. 2021, 14, 2201–2212. 10.2147/IDR.S306688. 34163185 PMC8214009

[ref3] Chinemerem Nwobodo D.; Ugwu M. C.; Oliseloke Anie C.; Al-Ouqaili M. T. S.; Chinedu Ikem J.; Victor Chigozie U.; Saki M. Antibiotic Resistance: The Challenges and Some Emerging Strategies for Tackling a Global Menace. Clin. Lab. Anal. 2022, 36 (9), e24655 10.1002/jcla.24655. PMC9459344 35949048

[ref4] Murugaiyan J.; Kumar P. A.; Rao G. S.; Iskandar K.; Hawser S.; Hays J. P.; Mohsen Y.; Adukkadukkam S.; Awuah W. A.; Jose R. A. M.; Sylvia N.; Nansubuga E. P.; Tilocca B.; Roncada P.; Roson-Calero N.; Moreno-Morales J.; Amin R.; Kumar B. K.; Kumar A.; Toufik A.-R.; Zaw T. N.; Akinwotu O. O.; Satyaseela M. P.; Van Dongen M. B. M. Progress in Alternative Strategies to Combat Antimicrobial Resistance: Focus on Antibiotics. Antibiotics 2022, 11 (2), 200 10.3390/antibiotics11020200. 35203804 PMC8868457

[ref5] Bruna T.; Maldonado-Bravo F.; Jara P.; Caro N. Silver Nanoparticles and Their Antibacterial Applications. Int. J. Mol. Sci. 2021, 22 (13), 7202 10.3390/ijms22137202. 34281254 PMC8268496

[ref6] Abdal Dayem A.; Hossain M.; Lee S.; Kim K.; Saha S.; Yang G.-M.; Choi H.; Cho S.-G. The Role of Reactive Oxygen Species (ROS) in the Biological Activities of Metallic Nanoparticles. Int. J. Mol. Sci. 2017, 18 (1), 120 10.3390/ijms18010120. 28075405 PMC5297754

[ref7] More P. R.; Pandit S.; Filippis A. D.; Franci G.; Mijakovic I.; Galdiero M. Silver Nanoparticles: Bactericidal and Mechanistic Approach against Drug Resistant Pathogens. Microorganisms 2023, 11 (2), 369 10.3390/microorganisms11020369. 36838334 PMC9961011

[ref8] Maklakova M.; Villarreal-Gómez L. J.; Nefedova E.; Shkil N.; Pestryakov A.; Bogdanchikova N. Role of Biofilm Formation in the Drop of Bacterial Resistance to Antibiotics after Animal Therapy with Silver Nanoparticles. ACS Appl. Nano Mater. 2024, 7 (14), 16553–16563. 10.1021/acsanm.4c02566.

[ref9] Garibo Ruiz D.; Nefedova E.; Shkil N. N.; Shkil N. A.; Vazquez-Gomez R. L.; Pestryakov A.; Bogdanchikova N. Silver Nanoparticles Targeting the Drug Resistance Problem of Streptococcus Dysgalactiae: Susceptibility to Antibiotics and Efflux Effect. Int. J. Mol. Sci. 2022, 23 (11), 6024 10.3390/ijms23116024. 35682703 PMC9181605

[ref10] Bogdanchikova N.; Maklakova M.; Villarreal-Gómez L. J.; Nefedova E.; Shkil N. N.; Plotnikov E.; Pestryakov A. Revealing the Second and the Third Causes of AgNPs Property to Restore the Bacterial Susceptibility to Antibiotics. Int. J. Mol. Sci. 2023, 24 (9), 7854 10.3390/ijms24097854. 37175561 PMC10178359

[ref11] Plotnikov E. V.; Tretayakova M. S.; Garibo-Ruíz D.; Rodríguez-Hernández A. G.; Pestryakov A. N.; Toledano-Magaña Y.; Bogdanchikova N. A Comparative Study of Cancer Cells Susceptibility to Silver Nanoparticles Produced by Electron Beam. Pharmaceutics 2023, 15 (3), 962 10.3390/pharmaceutics15030962. 36986823 PMC10056419

[ref12] Parvekar P.; Palaskar J.; Metgud S.; Maria R.; Dutta S. The Minimum Inhibitory Concentration (MIC) and Minimum Bactericidal Concentration (MBC) of Silver Nanoparticles against *Staphylococcus Aureus*. Biomater. Invest. Dentist. 2020, 7 (1), 105–109. 10.1080/26415275.2020.1796674. PMC7470068 32939454

[ref13] Clinical and Laboratory Standards Institute Performance Standards for Antimicrobial Susceptibility Testing. Malvern 2013 https://clsi.org/standards/products/microbiology/documents/m100/

[ref14] Kamimura R.; Kanematsu H.; Ogawa A.; Kogo T.; Miura H.; Kawai R.; Hirai N.; Kato T.; Yoshitake M.; Barry D. M. Quantitative Analyses of Biofilm by Using Crystal Violet Staining and Optical Reflection. Materials 2022, 15 (19), 6727 10.3390/ma15196727. 36234069 PMC9571847

[ref15] Baran A.; Kwiatkowska A.; Potocki L. Antibiotics and Bacterial Resistance—A Short Story of an Endless Arms Race. Int. J. Mol. Sci. 2023, 24 (6), 5777 10.3390/ijms24065777. 36982857 PMC10056106

[ref16] Huang X.; Li X.; An H.; Wang J.; Ding M.; Wang L.; Li L.; Ji Q.; Qu F.; Wang H.; Xu Y.; Lu X.; He Y.; Zhang J.-R. Capsule Type Defines the Capability of Klebsiella Pneumoniae in Evading Kupffer Cell Capture in the Liver. PLoS Pathog. 2022, 18 (8), e1010693 10.1371/journal.ppat.1010693. 35914009 PMC9342791

[ref17] Jajere S. M. A Review of Salmonella Enterica with Particular Focus on the Pathogenicity and Virulence Factors, Host Specificity and Antimicrobial Resistance Including Multidrug Resistance. Vet. World 2019, 12 (4), 504–521. 10.14202/vetworld.2019.504-521. 31190705 PMC6515828

[ref18] Kędziora A.; Wernecki M.; Korzekwa K.; Speruda M.; Gerasymchuk Y.; Łukowiak A.; Bugla-Płoskońska G. Consequences Of Long-Term Bacteria’s Exposure To Silver Nanoformulations With Different PhysicoChemical Properties. Int. J. Nanomed. 2020, 15, 199–213. 10.2147/IJN.S208838. PMC6970275 32021174

[ref19] Panáček A.; Kvítek L.; Smékalová M.; Večeřová R.; Kolář M.; Röderová M.; Dyčka F.; Šebela M.; Prucek R.; Tomanec O.; Zbořil R. Bacterial Resistance to Silver Nanoparticles and How to Overcome It. Nat. Nanotechnol. 2018, 13 (1), 65–71. 10.1038/s41565-017-0013-y. 29203912

[ref20] Stabryla L. M.; Johnston K. A.; Diemler N. A.; Cooper V. S.; Millstone J. E.; Haig S.-J.; Gilbertson L. M. Role of Bacterial Motility in Differential Resistance Mechanisms of Silver Nanoparticles and Silver Ions. Nat. Nanotechnol. 2021, 16 (9), 996–1003. 10.1038/s41565-021-00929-w. 34155383

[ref21] Khatoon N.; Alam H.; Khan A.; Raza K.; Sardar M. Ampicillin Silver Nanoformulations against Multidrug Resistant Bacteria. Sci. Rep. 2019, 9 (1), 6848 10.1038/s41598-019-43309-0. 31048721 PMC6497658

[ref22] Zhao H.; Wang M.; Cui Y.; Zhang C. Can We Arrest the Evolution of Antibiotic Resistance? The Differences between the Effects of Silver Nanoparticles and Silver Ions. Environ. Sci. Technol. 2022, 56 (8), 5090–5101. 10.1021/acs.est.2c00116. 35344362

[ref23] Wu K.; Li H.; Cui X.; Feng R.; Chen W.; Jiang Y.; Tang C.; Wang Y.; Wang Y.; Shen X.; Liu Y.; Lynch M.; Long H. Mutagenesis and Resistance Development of Bacteria Challenged by Silver Nanoparticles. Antimicrob. Agents Chemother. 2022, 66 (10), e0062822 10.1128/aac.00628-22. 36094196 PMC9578424

[ref24] Li J.; Yu L.; Wang R.; Lan J.; Li M.; Qiao Y.; Tao Z.; Lü H.; Wang F.; Fang Q.; Guo P. The Role of Silver Nanoparticles Alone and Combined with Imipenem on Carbapenem-Resistant *Klebsiella Pneumoniae*. J. Appl. Microbiol. 2024, 135 (5), lxae077 10.1093/jambio/lxae077. 38544327

[ref25] Graves J. L.; Tajkarimi M.; Cunningham Q.; Campbell A.; Nonga H.; Harrison S. H.; Barrick J. E. Rapid Evolution of Silver Nanoparticle Resistance in Escherichia Coli. Front. Genet. 2015, 6. 10.3389/fgene.2015.00042. PMC4330922 25741363

[ref26] Nefedova E.; Shkil N.; Shkil N.; Garibo D.; Luna Vazquez-Gomez R.; Pestryakov A.; Bogdanchikova N. Solution of the Drug Resistance Problem of Escherichia Coli with Silver Nanoparticles: Efflux Effect and Susceptibility to 31 Antibiotics. Nanomaterials 2023, 13 (6), 1088 10.3390/nano13061088. 36985982 PMC10054727

[ref27] Kaweeteerawat C.; Na Ubol P.; Sangmuang S.; Aueviriyavit S.; Maniratanachote R. Mechanisms of Antibiotic Resistance in Bacteria Mediated by Silver Nanoparticles. J. Toxicol. Environ. Health, Part A 2017, 80 (23–24), 1276–1289. 10.1080/15287394.2017.1376727. 29020531

[ref28] Nefedova E.; Shkil N.; Luna Vazquez-Gomez R.; Garibo D.; Pestryakov A.; Bogdanchikova N. AgNPs Targeting the Drug Resistance Problem of Staphylococcus Aureus: Susceptibility to Antibiotics and Efflux Effect. Pharmaceutics 2022, 14 (4), 763 10.3390/pharmaceutics14040763. 35456596 PMC9025349

